# Psychological factors associated with COVID-19 related anxiety and depression in young adults during the COVID-19 pandemic

**DOI:** 10.1371/journal.pone.0286636

**Published:** 2023-06-02

**Authors:** Ye Eun Lee, Jun Ho Seo, Shin Tae Kim, Sumoa Jeon, Chun Il Park, Se Joo Kim, Jee In Kang

**Affiliations:** 1 Institute of Behavioral Science in Medicine, Yonsei University College of Medicine, Seoul, Republic of Korea; 2 Department of Psychiatry, Yonsei university Wonju college of medicine, Wonju, Republic of Korea; 3 Department of Psychiatry, Yonsei University College of Medicine, Seoul, Republic of Korea; 4 Department of Psychiatry, CHA Bundang Medical Center, CHA University, Seongnam, Republic of Korea; University of Botswana, BOTSWANA

## Abstract

**Background:**

The coronavirus disease 2019 (COVID-19) pandemic and the corresponding lockdown have drastically changed our lives and led to high psychological distress and mental health problems. This study examined whether psychological factors such as loneliness, perfectionism, and health anxiety are associated with COVID-19 related anxiety and depression during the pandemic in young Korean adults, after controlling for various socio-demographic factors and early life stress.

**Methods:**

A total of 189 participants (58.2% women) completed a cross-sectional online survey including the Fear of COVID-19 Scale, Center for Epidemiologic Studies Depression Scale, 3-item Revised UCLA Loneliness Scale, Frost Multidimensional Perfectionism Scale, and Whiteley Index-6. Hierarchical linear regression analyses with three blocks were employed to identify the factors that contributed to COVID-19 related anxiety and depressive symptoms.

**Results:**

Hierarchical regression analyses showed that higher health anxiety was significantly associated with more severe COVID-19 related anxiety (standardized regression coefficient, β = 0.599, *p* < 0.001). Additionally, higher levels of loneliness (β = 0.482, *p* < 0.001), perfectionism (β = 0.124, *p* = 0.035), and health anxiety (β = 0.228, *p* < 0.001) were significantly associated with higher depression scores. The three psychological factors explained 32.8% of the total variance in depressive symptom scores, after taking all covariates into account.

**Conclusion:**

The results showed that health anxiety was a risk factor for both COVID-19 related anxiety and depression in young adults. Loneliness was the strongest predictor of depressive symptoms during the COVID-19 pandemic. These findings highlight the importance of identifying vulnerable individuals and encouraging psychological counselling and social connections to reduce the burden of psychiatric disorders during the pandemic.

## Introduction

Coronavirus disease 2019 (COVID-19) is an acute respiratory disease caused by the virus SARS-CoV-2. It started in Wuhan, China in December 2019 and spread worldwide as a pandemic. The outbreak has caused high levels of fear and worry about being infected. In addition, strategies to prevent the spread of COVID-19 have resulted in drastic changes and disruptions in our daily lives [1]. A recent systematic review reported an additional 76.2 million cases of anxiety disorder and 53.2 million cases of major depression worldwide caused by the pandemic in 2020 [2]. The pandemic has significantly impacted mental health in nearly half of the general population, and the burden of psychological morbidities was highest among patients with COVID-19 followed by healthcare workers and the general public [3].

Notably, the prevalence of anxiety and depression has risen dramatically among young adults [4], who may be exposed to more pandemic-related stressors such as campus closures, loss of income, and uncertainty about the future. Although young adults are particularly vulnerable to psychological distress and anxiety [5], they are least likely to seek psychological help during the pandemic [6]. Understanding the psychosocial factors that affect their poor mental health is important for the prevention of psychiatric disorders and targeted early intervention.

Various psychological factors that might be exacerbated due to the pandemic burden may play a major role in COVID-19 related anxiety and depression. Specifically, loneliness, a sense of feeling disconnected from others, and perceiving social isolation [7], may be an important risk factor of anxiety and depression during the COVID-19 pandemic. Based on Erikson’s theory of psychosocial development (1963), establishing intimate relationships is the main challenge for young adults between the ages of 18 and 40. Erikson believed that young adults who did not successfully achieve intimacy suffered isolation and loneliness, which could result in negative outcomes such as reduced ability to complete a subsequent stage [8]. As the COVID-19 pandemic has resulted in significantly increased loneliness due to physical distancing and quarantine [9] and the prevalence of loneliness is the highest in young adults [10], this may lead to detrimental effects on the mental health of young adults.

Another psychological factor that may influence negative health outcomes during the pandemic is perfectionism. Perfectionism, characterized by excessively high expectations and overly critical self-evaluations [11], is a relatively enduring, trait-like characteristic that is distinct from [12] and a known risk factor for depression and anxiety [13, 14]. A longitudinal study found that perfectionism is a vulnerability for, not a consequence of, depressive symptoms [15], and another study found that perfectionism predicts longitudinal increases in anxiety [16]. Maladaptive perfectionism, which is globally present among young adults at alarming levels, can have negative effects on mental health [17] and is associated with depressive and anxious symptoms in young adults [18]. Perfectionist young adults may especially have difficulty adapting to the uncertainty presented by stressful situations such as the COVID-19 pandemic as it disrupts their need for control [17]. Such loss of a sense of control has been suggested to be important in stress-diathesis understanding of perfectionism and depression [19], and perfectionists with a diminished sense of control are especially prone to anxiety [20].

In addition, health anxiety may be an important psychological factor affecting mental health during the pandemic. Health anxiety is inappropriate or excessive worrying about one’s health relative to the actual health status [21]. Since people with high health anxiety tend to be excessively aware of bodily sensations and interpret them as signs of a serious illness, they may react to health-related stress such as the pandemic with intense negative reactions [22] and have an increased risk of depression and anxiety [23]. Several studies have reported that health anxiety is associated with adverse mental health outcomes during the pandemic [24, 25].

Moreover, the COVID-19 pandemic affects mental health differently depending on socio-demographic characteristics [26], including age, sex, low socio-economic status (SES) [27, 28], and obesity [29]. In addition, early life stress, one of the most robust risk factors for anxiety and depression [30], is associated with high emotional distress in early adulthood during the pandemic [31]. Therefore, these factors need to be considered as potential confounders to explore a clear relationship between certain psychological factors and mental health problems during the pandemic.

This study identified potential psychological factors such as loneliness, perfectionism, and health anxiety that contribute to COVID-19 related anxiety and depression in young adults, who may be more vulnerable to psychosocial distress during the pandemic, after controlling for various socio-demographic factors and early life stress.

## Materials and methods

### Ethics statement

The study protocol was approved by the Institutional Review Board of Severance Hospital. The IRB approval number was 4-2021-0542. The participants provided online consent prior to the start of this study.

### Participants

A total of 189 participants were recruited through a notice posted on the web survey site Survey Monkey from July to November 2021. All the participants were between 18 and 40 years of age and understood the purpose and process of the research. The exclusion criteria were as follows: (1) self-reported history of psychiatric disorders, (2) history of brain injury, convulsive disorders, or any neurological symptoms, and (3) physical or mental illness that could interfere with the completion of the survey. The participants were paid for their participation (approximately $25).

### Measures

#### Socio-demographic characteristics

Socio-demographic variables were age, sex, height, weight, SES, and education level. SES was the following six categories: 1) very low, 2) low, 3) middle, 4) middle to high, 5) high, and 6) very high. Education level was classified into two: (1) < high school and (2) ≥ high school.

#### Fear of COVID-19 Scale (FCV-19S)

COVID-19 related anxiety was assessed using the Fear of COVID-19 Scale (FCV-19S), a newly developed self-report tool to assess fear of COVID-19 [32]. The FCV-19S consists of seven items (e.g., item 1, “I am most afraid of Corona,” and item 7, “My heart races or palpitates when I think about getting Corona.”) related to emotional fear reactions specific to the COVID-19 pandemic. Respondents were asked to rate their level of agreement with each item on a 5-point Likert scale (1  =  strongly disagree to 5  =  strongly agree). Scores ranged from 7 to 35, with higher overall scores indicating a more severe fear of COVID-19. The initial validation study showed robust psychometric properties, with good validity and reliability in assessing fear of COVID-19 among the general population [32]. The validity and reliability of the Korean version have been verified, and the measured fear shows a significant correlation with anxiety [33]. The internal consistency of the present sample was 0.85.

#### Center for Epidemiologic Studies Depression Scale (CES-D)

Depression was assessed using the Center for Epidemiologic Studies Depression Scale (CES-D), which was designed to measure depressive symptoms in the general population [34]. It is a self-report scale consisting of 20 items and measures depressive mood over the past seven days on a 4-point Likert scale ranging from 0 to 3. The scores ranged from 0 to 60, with higher scores indicating more severe depressive symptoms. The scale has demonstrated high internal consistency and good validity across different demographic characteristics in the general population [34]. The Korean version has shown high reliability and validity in clinical and nonclinical populations [35]. The internal consistency of the present sample was 0.77.

#### Early Trauma Inventory Self-Report-Short Form (ETISR-SF)

Early life stress was assessed using the Early Trauma Inventory Self Report-Short Form (ETISR-SF) [36], a self-report scale consisting of 27 items answered as ‘yes’ (coded as 1) or ‘no’ (coded as 0). The scores ranged from 0 to 27, with higher scores indicating the occurrence of more traumatic events before the age of 18. This measure was found to have good validity and internal consistency in individuals with and without psychiatric disorders [36] and has shown excellent validity and internal consistency among Korean adults [37]. The internal consistency of the present sample was 0.81.

#### 3-item Revised UCLA Loneliness Scale

Perceived loneliness was assessed using three items from the 20-item Revised UCLA Loneliness Scale, version 3 (RULS). The three items, adapted based on previous studies using factor analyses [38], were: (1) lack of companionship, (2) felt left out, and (3) felt isolated in the past two weeks. The 4-point Likert scale (1 = never, 2 = rarely, 3 = sometimes, and 4 = always) that was used in the RULS was adopted. Scores ranged from 3 to 12, with higher scores indicating higher levels of loneliness. The original RULS has shown high reliability and validity [39], and the Korean version of RULS has demonstrated high validity and reliability with an internal consistency of 0.93 [40]. The internal consistency of the present sample was 0.88.

#### Frost Multidimensional Perfectionism Scale (FMPS)

Perfectionism was assessed using the Frost Multidimensional Perfectionism Scale (FMPS) [11], a self-report scale composed of 35 items rated on a 5-point Likert scale ranging from 1 to 5. The scores ranged from 35 to 175, with higher scores indicating higher levels of perfectionism. The scale has shown good internal consistency and convergent validity within clinical and nonclinical samples [11]. The Korean version has demonstrated construct and predictive validity [41]. The internal consistency of the present sample was 0.92.

#### Whiteley Index-6 (WI-6)

Health anxiety was assessed using the Whiteley Index-6 (WI-6) [42], a self-report scale composed of six items rated on a 5-point Likert scale ranging from 0 to 4. The WI scale was designed for measuring hypochondriacal concerns of health worry (e.g., “worry a lot about your health”), disease conviction (e.g.,” hard for you to believe the doctor when he or she tells you there is nothing to worry about”), bodily preoccupation (e.g., “bothered by many different symptoms”). The scores ranged from 0 to 24, with higher scores indicating higher levels of health anxiety. The scale has shown superior stability and good internal consistency [42]. The Whiteley Index has been used to measure health anxiety in Korean university students with adequate reliability [43, 44]. The internal consistency of the present sample was 0.85.

### Data analysis

The sample characteristics were determined using descriptive statistics. Pearson’s correlations were conducted to examine the relationships between demographic characteristics, early life stress, psychological factors, and COVID-19 related anxiety and depression. To determine whether loneliness, perfectionism, and health anxiety were related to COVID-19 related anxiety and depression, hierarchical multiple linear regression analyses were conducted with COVID-19 related anxiety and depression as the dependent variable. Based on evidence from the existing literature, factors that might contribute to anxiety and depression were determined and selected as independent variables of the regression model, and they were grouped into three blocks. Block 1 included the socio-demographic variables (age, sex, body mass index, SES, and education); Block 2, early life stress (ETISR-SF); Block 3, the psychological variables (3-item RULS, FMPS, and WI-6), which were our key variables of interest to examine their unbiased associations with COVID-19 related anxiety (FCV-19S) and depression (CES-D). To exclude multicollinearity between the independent variables, the variance inflation factor (VIF) for each variable was computed in the full model. VIF values less than 2.5 were signs of no serious multicollinearity issues. In the full model, the VIF values for all explanatory variables were in the range of 1.014–1.462. Statistical significance was set at *p* < 0.05. All statistical analyses were conducted using Statistical Package for the Social Sciences version 26 (SPSS Inc., Chicago, IL, USA).

## Results

The socio-demographic and psychological characteristics of the participants are presented in Table 1. Pearson’s correlations showed that SES was significantly and negatively correlated with CES-D (*p* = 0.011). In addition, ETISR-SF and psychological factors (3-item RULS, FMPS, and WI-6) were positively associated with FCV-19S and CES-D scores (Table 2).

**Table 1:** Descriptive statistics.

Participants	N = 189
Age, years	27.74 ± 5.33 (19 ~ 40)
Sex	
Male	79 (41.8)
Female	110 (58.2)
BMI, kg/m^2^	22.91 ± 4.43
Education	
< High school	18 (9.5)
≥ High school	171 (90.5)
SES	
Very low	4 (2.1)
Low	52 (27.5)
Middle	105 (55.6)
Middle to high	21 (11.1)
High	6 (3.2)
Very high	1 (0.5)
ETISR-SF	4.63 ± 3.87
3-item RULS	5.73 ± 2.48
FMPS	99.45 ± 20.91
WI-6	6.14 ± 4.46

BMI, body mass index; SES, socio-economic status; ETISR-SF, Early Trauma Inventory Self-Report-Short Form; RULS, Revised UCLA Loneliness Scale; FMPS, Frost Multidimensional Perfectionism Scale; WI-6, Whiteley Index-6. Values are expressed as the mean ± standard deviation or n (%).

**Table 2:** Pearson’s correlation coefficients between psychological factors and COVID-19 related anxiety and depression (n = 189).

Predictor	FCV-19S		CES-D	
	*r*	*p*	*r*	*P*
ETISR-SF	0.235	0.001	0.404	<0.001
3-item RULS	0.253	<0.001	0.659	<0.001
FMPS	0.265	<0.001	0.413	<0.001
WI-6	0.643	<0.001	0.472	<0.001

FCV-19S, Fear of COVID-19 Scale; CES-D, Center for Epidemiologic Studies Depression Scale; ETISR-SF, Early Trauma Inventory Self-Report-Short Form; RULS, Revised UCLA Loneliness Scale; FMPS, Frost Multidimensional Perfectionism Scale; WI-6, Whiteley Index-6.

Hierarchical linear regression analyses were conducted with the three blocks. Table 3 shows the results of regression analyses using the three models for COVID-19 related anxiety. In Model 1, women scored significantly higher on the FCV-19S (standardized regression coefficient, β = 0.197, *p* = 0.011). In Model 2, higher ETISR-SF scores were significantly associated with higher FCV-19S scores. Early life stress added 4.4% to the variance explanation (increment in adjusted R^2^). In Model 3, higher health anxiety based on the WI-6 (β = 0.599, *p* < 0.001) was associated with higher FCV-19S scores (Table 2). The final model explained 41.1% of the variance in FCV-19S sum score.

**Table 3:** Hierarchical regression analysis of the relationship between psychological factors and COVID-19 related anxiety after controlling for socio-demographic variables and early life stress (n = 189).

Predictor	Model 1			Model 2			Model 3		
	*R*^*2*^ = .032, Δ*R*^*2*^ = .032			*R*^*2*^ = .076, Δ*R*^*2*^ = .044**			*R*^*2*^ = .411, Δ*R*^*2*^ = .335***		
	*B*	*SE B*	*β*	*B*	*SE B*	*β*	*B*	*SE B*	*β*
Constant	-2.350			-3.513			-4.364		
**Block 1: socio-demographic variables**									
Age	0.071	0.056	0.091	0.044	0.056	0.057	-0.006	0.046	-0.008
Sex	1.642	0.638	0.197*	1.720	0.624	0.206**	1.145	0.506	0.137*
BMI	0.120	0.072	0.129	0.123	0.070	0.132	0.081	0.056	0.087
SES	-0.555	0.380	-0.107	-0.442	0.372	-0.085	-0.152	0.310	-0.029
Education	0.102	1.011	0.007	0.389	0.992	0.028	0.343	0.796	0.024
**Block 2: early life stress**									
ETISR-SF				0.240	0.077	0.226**	0.089	0.069	0.083
**Block 3: psychological variables**									
3-item RULS							-0.019	0.112	-0.012
FMPS							0.007	0.013	0.038
WI-6							0.554	0.058	0.599***

Note. *N* = 189. Total *R*^*2*^ = .382. BMI, body mass index; SES, socio-economic status; ETISR-SF, Early Trauma Inventory Self-Report-Short Form; RULS, Revised UCLA Loneliness Scale; FMPS, Frost Multidimensional Perfectionism Scale; WI-6, Whiteley Index-6.

*p < .05.

**p < .01.

***p < .001.

On the contrary, Table 4 shows the results for the regression analyses with the three models for depressive symptoms. In Model 1, sex (female) (β = 0.250, *p* = 0.001) and lower SES (β = - 0.191, *p* = 0.009) were significantly associated with higher CES-D scores. In Model 2, higher ETISR-SF scores (β = 0.394, *p* < 0.001) were significantly associated with higher CES-D scores. Early life stress added 14.7% to the variance explanation (increment in adjusted R^2^). In Model 3, higher levels of 3-item RULS (β = 0.482, p < 0.001), FMPS (β = 0.124, *p* = 0.035), and WI-6 (β = 0.228, *p* < 0.001) were significantly associated with higher CES-D scores (Table 3). The three psychological factors explained 32.8% of the total variance in depressive symptom scores, after taking all covariates into account. The final model explained 54.6% of the variance in CES-D sum score.

**Table 4:** Hierarchical regression analysis of the relationship between psychological factors and depression after controlling for socio-demographic variables and early life stress (n = 189).

Predictor	Model 1			Model 2			Model 3		
	*R*^*2*^ = .071, Δ*R*^*2*^ = .071**			*R*^*2*^ = .218, Δ*R*^*2*^ = .147***			*R*^*2*^ = .546, Δ*R*^*2*^ = .328***		
	*B*	*SE B*	*β*	*B*	*SE B*	*β*	*B*	*SE B*	*β*
Constant	9.496			4.434			-3.204		
**Block 1: socio-demographic variables**									
Age	0.126	0.138	0.066	0.010	0.128	0.005	-0.146	0.100	-0.076
Sex	5.177	1.557	0.250**	5.517	1.429	0.266***	3.331	1.106	0.161**
BMI	0.190	0.175	0.082	0.206	0.161	0.089	0.143	0.123	0.061
SES	-2.457	0.926	-0.191**	-1.968	0.853	-0.153*	-1.586	0.678	-0.123*
Education	-1.387	2.467	-0.040	-0.135	2.273	-0.004	-1.513	1.741	-0.043
**Block 2: early life stress**									
ETISR-SF				1.045	0.175	0.394***	0.256	0.152	0.096
**Block 3: psychological variables**									
3-item RULS							1.990	0.246	0.482***
FMPS							0.061	0.029	0.124*
WI-6							0.525	0.127	0.228***

Note. *N* = 189. Total *R*^*2*^ = .555. BMI, body mass index; SES, socio-economic status; ETISR-SF, Early Trauma Inventory Self-Report-Short Form; RULS, Revised UCLA Loneliness Scale; FMPS, Frost Multidimensional Perfectionism Scale; WI-6, Whiteley Index-6.

*p < .05.

**p < .01.

***p < .001.

## Discussion

This study examined whether loneliness, perfectionism, and health anxiety were related to COVID-19 related anxiety and depression, after controlling for various socio-demographic factors and early life stress. Hierarchical regression showed that sex (female), early life stress, and high health anxiety were significant independent factors for COVID-19 related anxiety. In addition, sex (female), SES, early life stress, and the three psychological factors of loneliness, perfectionism, and health anxiety were significant independent factors for depressive symptoms during the COVID-19 pandemic.

Among the psychological factors, health anxiety was the only factor associated with both COVID-19 related anxiety and depression. These results suggest that health anxiety contributes to COVID-19 related mental health problems such as anxiety and depression in young adults. Consistent with our finding, recent studies suggest that health anxiety plays a crucial role in adverse mental health outcomes in the midst of the pandemic. Several studies have shown that increase in anxiety about COVID-19 is greater for health anxious individuals [45] including young adults in different countries [46, 47]. A previous study by our group showed that health anxiety was the most important symptom that connected COVID-19 anxiety to other clinical psychopathologies in non-psychotic psychiatric outpatients [48]. Those who have health anxiety show excessive fear of having a disease, which leads to increased vigilance and a vicious cycle of increased anxiety [49]. During the current pandemic, individuals are exposed to large amounts of information regarding COVID-19 through media outlets, which intensifies health anxiety among vulnerable individuals [21] such as young adults who report significant health anxiety during the pandemic [50]. As health anxiety is a concern for decision makers, health authorities, and healthcare professionals, further studies are needed to examine individual differences in health anxiety and their impact on mental health and behavior in the context of COVID-19.

In the hierarchical regression model for depression, loneliness was the strongest predictor of depressive symptoms. Recent studies have shown that loneliness during the COVID-19 pandemic is an important factor associated with depressive symptoms [51, 52]. Loneliness shows a strong association with mental health during the pandemic in young adults across different continents [53, 54]. The pandemic is a time of considerable loneliness due to the physical and social restrictions put into place to prevent the spread of the virus, and lonely individuals may be especially vulnerable to negative mental health outcomes. In particular, young adults were shown to be more prone to loneliness [55] and disproportionately impacted by it during the pandemic [56]. Recent studies show that there has been an increase in loneliness among young people during the COVID-19 pandemic, which is associated with an increase in depression [57]. The consequences of social isolation resulting from the pandemic may have especially significant implications for young adults, and our results suggest that lonely young adults may be more vulnerable to depression during the pandemic. As loneliness is a preventable public health issue, encouraging social connections is helpful for vulnerable people during the pandemic.

In addition, perfectionistic traits were a predictor of higher depressive symptoms during the pandemic in the regression model for depression. This finding is consistent with other studies among different countries [58, 59]. A recent Portuguese study reported that perfectionism is a vulnerable trait associated with psychological reactions and distress to the pandemic [58]. Considering that perfectionists have difficulties with diminished sense of control during uncertain situations [17], they may be especially vulnerable to psychiatric disorders such as depression and anxiety when faced with various unexpected situations, threats to safety, and disruptions in daily routines during the COVID-19 pandemic. A meta-analytic test of the Perfectionism Social Disconnection Model showed that perfectionism confers risk for depression via social disconnection [60]. Additionally, as perfectionists tend to be achievement-oriented [17], limited opportunities during the pandemic may lead to high levels of distress experienced by young adults with highly perfectionistic tendencies.

On the other hand, loneliness and perfectionism were not significantly associated with COVID-19 related anxiety measured by the FCV-19S in the regression model, although there was a weak positive correlation between the FCV-19S score and loneliness (*r* = 0.253, *p* < 0.001), and perfectionism (*r* = 0.265, *p* < 0.001) in the Pearson correlation analysis. A possible explanation is that the main concerns of people with high levels of loneliness or perfectionism are more likely to be related to other dimensions of anxiety, such as social and generalized anxiety in the context of the pandemic, rather than to the specific fear of COVID-19 itself.

There are several limitations that should be noted in this study. First, because it was cross-sectional, the levels of depression with pre-COVID-19 data could not be compared, and a causal relationship between the related factors and depression could not be determined. Second, the present data based on various self-reports obtained cross-sectionally may have difficulty in clearly distinguishing anxiety and depression from other psychological risk factors, although each scale was designed to assess a specific aspect of psychological factors or psychopathologies. Therefore, longitudinal studies using data combining self-reports and objective findings may better understand the nature of depression and its risk factors for young adults during the pandemic. Third, there may be potential confounders that could affect COVID-19 related anxiety and depression, which were not accounted for in this study. Fourth, the sample size of this study was relatively small. Replication studies with a larger sample size and longitudinal follow-up studies are needed to understand the relationships among mental health, psychological factors, and lifestyle changes that may be affected by the pandemic in young adults. Finally, as the participants of this study were young Korean adults, the present findings cannot be generalized to other populations.

In conclusion, the results showed that health anxiety was a risk factor for both COVID-19 related anxiety and depression in young adults. Loneliness was the strongest predictor of depressive symptoms during the COVID-19 pandemic. The findings highlight the importance of identifying vulnerable individuals during the pandemic and encouraging them for psychological counselling and social connections to reduce the burden of psychiatric disorders such as anxiety and depression and promote mental health. The present study can contribute to inform policies and interventions to screen for vulnerable individuals during the pandemic. Increased effort should be made to understand the needs of vulnerable young adults. An increase in investment, quality, and access to mental health services is needed to mitigate the effects of the COVID-19 pandemic on mental health and to provide support for young adults at risk.

## Supporting information

S1 Table Model summary for the hierarchical regression analysis of the relationship between psychological factors and COVID-19 related anxiety after controlling for socio-demographic variables and early life stress (n = 189). (DOCX)

S2 Table Hierarchical regression analysis of the relationship between psychological factors and COVID-19 related anxiety after controlling for socio-demographic variables and early life stress (n = 189). (DOCX)

S3 Table Model summary for the hierarchical regression analysis of the relationship between psychological factors and depression after controlling for socio-demographic variables and early life stress (n = 189). (DOCX)

S4 Table Hierarchical regression analysis of the relationship between psychological factors and depression after controlling for socio-demographic variables and early life stress (n = 189). (DOCX)

S1 File. Dataset (XLSX)

## References

[1] Giuntella O, Hyde K, Saccardo S, Sadoff S. Lifestyle and mental health disruptions during COVID-19. Proc Natl Acad Sci. 2021;118(9):e2016632118. doi: 10.1073/pnas.2016632118 33571107 PMC7936339

[2] Santomauro DF, Mantilla Herrera AM, Shadid J, Zheng P, Ashbaugh C, Pigott DM, et al. Global prevalence and burden of depressive and anxiety disorders in 204 countries and territories in 2020 due to the COVID-19 pandemic. Lancet. 2021;398(10312):1700–12. doi: 10.1016/S0140-6736(21)02143-7 34634250 PMC8500697

[3] Krishnamoorthy Y, Nagarajan R, Saya GK, Menon V. Prevalence of psychological morbidities among general population, healthcare workers and COVID-19 patients amidst the COVID-19 pandemic: a systematic review and meta-analysis. Psychiatry Res. 2020;293:113382. doi: 10.1016/j.psychres.2020.113382 32829073 PMC7417292

[4] Liu CH, Zhang E, Wong GTF, Hyun S, Hahm HC. Factors associated with depression, anxiety, and PTSD symptomatology during the COVID-19 pandemic: clinical implications for U.S. young adult mental health. Psychiatry Res. 2020;290:113172–. doi: 10.1016/j.psychres.2020.113172 32512357 PMC7263263

[5] Rossell SL, Neill E, Phillipou A, Tan EJ, Toh WL, Van Rheenen TE, et al. An overview of current mental health in the general population of Australia during the COVID-19 pandemic: results from the COLLATE project. Psychiatry Res. 2021;296:113660-. doi: 10.1016/j.psychres.2020.113660 33373808 PMC7836867

[6] Glowacz F, Schmits E. Psychological distress during the COVID-19 lockdown: the young adults most at risk. Psychiatry Res. 2020;293:113486. doi: 10.1016/j.psychres.2020.113486 33007682 PMC7518205

[7] Hawkley LC, Cacioppo JT. Loneliness matters: a theoretical and empirical review of consequences and mechanisms. Ann Behav Med. 2010;40(2):218–27. doi: 10.1007/s12160-010-9210-8 20652462 PMC3874845

[8] Erikson EH. Childhood and Society1963.

[9] Killgore WDS, Cloonan SA, Taylor EC, Dailey NS. Loneliness: a signature mental health concern in the era of COVID-19. Psychiatry Res. 2020;290:113117. doi: 10.1016/j.psychres.2020.113117 32480121 PMC7255345

[10] Cigna International. Cigna U.S. loneliness index 2018 [Available from: https://www.cigna.com/static/www-cigna-com/docs/about-us/newsroom/studies-and-reports/combatting-loneliness/loneliness-survey-2018-full-report.pdf.

[11] Frost RO, Marten P, Lahart C, Rosenblate R. The dimensions of perfectionism. Cognit Ther Res. 1990;14(5):449–68.

[12] Mackinnon SP, Battista SR, Sherry SB, Stewart SH. Perfectionistic self-presentation predicts social anxiety using daily diary methods. Pers Individ Dif. 2014;56:143–8.

[13] Chang EC. Perfectionism and loneliness as predictors of depressive and anxious symptoms in Asian and European Americans: do self-construal schemas also matter? Cognit Ther Res. 2013;37:1179–88.

[14] Chang EC, Hirsch JK, Sanna LJ, Jeglic EL, Fabian CG. A preliminary study of perfectionism and loneliness as predictors of depressive and anxious symptoms in Latinas: a top-down test of a model. J Couns Psychol. 2011;58(3):441. doi: 10.1037/a0023255 21517153

[15] Sherry SB, Richards JE, Sherry DL, Stewart SH. Self-critical perfectionism is a vulnerability factor for depression but not anxiety: a 12-month, 3-wave longitudinal study. J Res Pers. 2014;52:1–5.

[16] Smith MM, Vidovic V, Sherry SB, Stewart SH, Saklofske DH. Are perfectionism dimensions risk factors for anxiety symptoms? A meta-analysis of 11 longitudinal studies. Anxiety Stress Coping. 2018;31(1):4–20. doi: 10.1080/10615806.2017.1384466 28980487

[17] Flett GL, Hewitt PL. The perfectionism pandemic meets COVID-19: understanding the stress, distress, and problems in living for perfectionists during the Global Health crisis. J Concurrent Disord. 2020;2:80–105.

[18] Doyle I, Catling JC. The influence of perfectionism, self-esteem and resilience on young people’s mental health. J Psychol. 2022;156(3):224–40. doi: 10.1080/00223980.2022.2027854 35201966

[19] Hewitt PL, Flett GL. Dimensions of perfectionism, daily stress, and depression: a test of the specific vulnerability hypothesis. J Abnorm Psychol. 1993;102(1):58–65. doi: 10.1037//0021-843x.102.1.58 8436700

[20] Mor S, Day HI, Flett GL, Hewitt PL. Perfectionism, control, and components of performance anxiety in professional artists. Cognit Ther Res. 1995;19(2):207–25.

[21] Asmundson GJG, Abramowitz JS, Richter AA, Whedon M. Health anxiety: current perspectives and future directions. Curr Psychiatry Rep. 2010;12(4):306–12. doi: 10.1007/s11920-010-0123-9 20549396

[22] Taylor S. The Psychology of Pandemics: Preparing for the Next Global Outbreak of Infectious Disease: Cambridge Scholars Publishing; 2019.

[23] Sunderland M, Newby JM, Andrews G. Health anxiety in Australia: prevalence, comorbidity, disability and service use. Br J Psychiatry. 2013;202(1):56–61. doi: 10.1192/bjp.bp.111.103960 22500013

[24] Landi G, Pakenham KI, Boccolini G, Grandi S, Tossani E. Health anxiety and mental health outcome during COVID-19 lockdown in Italy: the mediating and moderating roles of psychological flexibility. Front Psychol. 2020;11. doi: 10.3389/fpsyg.2020.02195 32982888 PMC7488226

[25] Nikčević AV, Marino C, Kolubinski DC, Leach D, Spada MM. Modelling the contribution of the Big Five personality traits, health anxiety, and COVID-19 psychological distress to generalised anxiety and depressive symptoms during the COVID-19 pandemic. J Affect Disord. 2021;279:578–84. doi: 10.1016/j.jad.2020.10.053 33152562 PMC7598311

[26] Caycho-Rodríguez T, Tomás JM, Vilca LW, Carbajal-León C, Cervigni M, Gallegos M, et al. Socio-Demographic Variables, Fear of COVID-19, Anxiety, and Depression: Prevalence, Relationships and Explanatory Model in the General Population of Seven Latin American Countries. Front Psychol. 2021;12:695989. doi: 10.3389/fpsyg.2021.695989 34803794 PMC8602858

[27] Pieh C, Budimir S, Probst T. The effect of age, gender, income, work, and physical activity on mental health during Coronavirus disease (COVID-19) lockdown in Austria. J Psychosom Res. 2020;136:110186. doi: 10.1016/j.jpsychores.2020.110186 32682159 PMC7832650

[28] Wang Y, Kala MP, Jafar TH. Factors associated with psychological distress during the coronavirus disease 2019 (COVID-19) pandemic on the predominantly general population: A systematic review and meta-analysis. PLoS One. 2020;15(12):e0244630. doi: 10.1371/journal.pone.0244630 33370404 PMC7769562

[29] Melamed OC, Selby P, Taylor VH. Mental Health and Obesity During the COVID-19 Pandemic. Curr Obes Rep. 2022;11(1):23–31. doi: 10.1007/s13679-021-00466-6 35254633 PMC8899440

[30] Nugent NR, Tyrka AR, Carpenter LL, Price LH. Gene-environment interactions: early life stress and risk for depressive and anxiety disorders. Psychopharmacology (Berl). 2011;214(1):175–96. doi: 10.1007/s00213-010-2151-x 21225419 PMC3615637

[31] Li X, Lv Q, Tang W, Deng W, Zhao L, Meng Y, et al. Psychological stresses among Chinese university students during the COVID-19 epidemic: the effect of early life adversity on emotional distress. J Affect Disord. 2021;282:33–8. doi: 10.1016/j.jad.2020.12.126 33387744 PMC7985596

[32] Ahorsu DK, Lin C-Y, Imani V, Saffari M, Griffiths MD, Pakpour AH. The fear of COVID-19 scale: development and initial validation. Int J Ment Health Addict. 2020:1–9. 10.1007/s11469-020-00270-8 PMC7100496 32226353

[33] Han J-W, Park J, Lee H. Validity and reliability of the Korean version of the Fear of COVID-19 Scale. Int J Environ Res Public Health. 2021;18(14):7402. doi: 10.3390/ijerph18147402 34299854 PMC8306206

[34] Radloff LS. The CES-D scale: a self-report depression scale for research in the general population. Appl Psychol Meas. 1977;1(3):385–401.

[35] Cho MJ, Kim KH. Use of the Center for Epidemiologic Studies Depression (CES-D) scale in Korea. J Nerv Ment Dis. 1998;186(5):304–10. doi: 10.1097/00005053-199805000-00007 9612448

[36] Bremner JD, Bolus R, Mayer EA. Psychometric properties of the Early Trauma Inventory-Self Report. J Nerv Ment Dis. 2007;195(3):211–8. doi: 10.1097/01.nmd.0000243824.84651.6c 17468680 PMC3229091

[37] Jeon J-R, Lee E-H, Lee S-W, Jeong E-g, Kim J-H, Lee D, et al. The Early Trauma Inventory Self Report-Short Form: psychometric properties of the korean version. Psychiatry Investig. 2012;9(3):229–35. doi: 10.4306/pi.2012.9.3.229 22993521 PMC3440471

[38] Hughes ME, Waite LJ, Hawkley LC, Cacioppo JT. A short scale for measuring loneliness in large surveys: results from two population-based studies. Res Aging. 2004;26(6):655–72. doi: 10.1177/0164027504268574 18504506 PMC2394670

[39] Russell D, Peplau LA, Cutrona CE. The revised UCLA Loneliness Scale: concurrent and discriminant validity evidence. J Pers Soc Psychol. 1980;39(3):472–80. doi: 10.1037//0022-3514.39.3.472 7431205

[40] Kim OS. Korean version of the revised UCLA loneliness scale: reliability and validity test. J Nurs Acad Soc. 1997;27(4):871–9.

[41] Chung SJ, Yon MH. A study of the development of cognitive-behavioral group counseling program for reducing the perfectionism. Korean J Counsel Psychotherapy. 2000;12(2):147–67.

[42] Asmundson GJG, Carleton NR, Bovell CV, Taylor S. Comparison of unitary and multidimensional models of the Whiteley Index in a nonclinical sample: implications for understanding and assessing health anxiety. J Cogn Psychother. 2008(2):87–96.

[43] Yi IH. Factor structure of the Illness Attitudes Scale(IAS) in a Korean college sample. Kor J Psychol Health. 2004;9(1):203–18.

[44] Shin HK, Won HT. A study on the development of the Korean Alexithymia Scale. Kor J Psychol Health. 1997;16(2):219–31.

[45] Asmundson GJG, Taylor S. How health anxiety influences responses to viral outbreaks like COVID-19: what all decision-makers, health authorities, and health care professionals need to know. J Anxiety Disord. 2020;71:102211. doi: 10.1016/j.janxdis.2020.102211 32179380 PMC7271220

[46] Jungmann SM, Witthöft M. Health anxiety, cyberchondria, and coping in the current COVID-19 pandemic: which factors are related to coronavirus anxiety? J Anxiety Disord. 2020;73:102239. doi: 10.1016/j.janxdis.2020.102239 32502806 PMC7239023

[47] Canlı D, Karaşar B. Health anxiety and emotion regulation during the period of COVID-19 outbreak in Turkey. Psychiatr Danub. 2020;32(3–4):513–20. doi: 10.24869/psyd.2020.513 33370761

[48] Kim ST, Seo JH, Lee S, Jeon S, Park CI, Kim SJ, et al. Dysfunctional Coronavirus anxiety in nonpsychotic psychiatric outpatients during the COVID-19 pandemic: a network analysis. Depress Anxiety. 2022. doi: 10.1002/da.23256 35344625 PMC9087144

[49] Tyrer P. COVID-19 health anxiety. World Psychiatry 2020;19(3):307–8. doi: 10.1002/wps.20798 32931105 PMC7491644

[50] Kibbey MM, Fedorenko EJ, Farris SG. Anxiety, depression, and health anxiety in undergraduate students living in initial US outbreak “hotspot” during COVID-19 pandemic. Cogn Behav Ther. 2021;50(5):409–21. doi: 10.1080/16506073.2020.1853805 33433271

[51] Okruszek Ł, Aniszewska-Stańczuk A, Piejka A, Wiśniewska M, Żurek K. Safe but lonely? Loneliness, anxiety, and depression symptoms and COVID-19. Front Psychol. 2020;11. doi: 10.3389/fpsyg.2020.579181 33343454 PMC7747668

[52] Palgi Y, Shrira A, Ring L, Bodner E, Avidor S, Bergman Y, et al. The loneliness pandemic: loneliness and other concomitants of depression, anxiety and their comorbidity during the COVID-19 outbreak. J Affect Disord. 2020;275:109–11. doi: 10.1016/j.jad.2020.06.036 32658811 PMC7330569

[53] Horigian VE, Schmidt RD, Feaster DJ. Loneliness, mental health, and substance use among US young adults during COVID-19. J Psychoactive Drugs. 2021;53(1):1–9. doi: 10.1080/02791072.2020.1836435 33111650

[54] Werner AM, Tibubos AN, Mülder LM, Reichel JL, Schäfer M, Heller S, et al. The impact of lockdown stress and loneliness during the COVID-19 pandemic on mental health among university students in Germany. Sci Rep. 2021;11(1):22637. doi: 10.1038/s41598-021-02024-5 34811422 PMC8609027

[55] Shovestul B, Han J, Germine L, Dodell-Feder D. Risk factors for loneliness: the high relative importance of age versus other factors. PLoS One. 2020;15(2):e0229087. doi: 10.1371/journal.pone.0229087 32045467 PMC7012443

[56] Wickens CM, McDonald AJ, Elton-Marshall T, Wells S, Nigatu YT, Jankowicz D, et al. Loneliness in the COVID-19 pandemic: associations with age, gender and their interaction. J Psychiatr Res. 2021;136:103–8. doi: 10.1016/j.jpsychires.2021.01.047 33582608 PMC8635289

[57] Lee CM, Cadigan JM, Rhew IC. Increases in loneliness among young adults during the COVID-19 pandemic and association with increases in mental health problems. J Adolesc Health. 2020;67(5):714–7. doi: 10.1016/j.jadohealth.2020.08.009 33099414 PMC7576375

[58] Pereira AT, Cabaços C, Araújo A, Amaral AP, Carvalho F, Macedo A. COVID-19 psychological impact: the role of perfectionism. Pers Individ Dif. 2022;184:111160. doi: 10.1016/j.paid.2021.111160 34642518 PMC8496947

[59] Molnar DS, Methot-Jones T, Moore J, O’Leary DD, Wade TJ. Perfectionistic cognitions pre-pandemic predict greater anxiety symptoms during the pandemic among emerging adults: a two-wave cross-lagged study. J Ration Emot Cogn Behav Ther. 2021:1–19. doi: 10.1007/s10942-021-00423-1 34690428 PMC8527291

[60] Smith MM, Sherry SB, Vidovic V, Hewitt PL, Flett GL. Why does perfectionism confer risk for depressive symptoms? A meta-analytic test of the mediating role of stress and social disconnection. Journal of Research in Personality. 2020;86:103954.

