# Supplementary material for: Psychological factors associated with COVID-19 related anxiety and depression in young adults during the COVID-19 pandemic
Source: PLoS One. 2023 Jun 2;18(6):e0286636. doi: 10.1371/journal.pone.0286636 (PMC10237641; doi:10.1371/journal.pone.0286636)
Supplement: S1 Table — (DOCX) [file pone.0286636.s001.docx]

**S1 Table. Model summary for the hierarchical regression analysis of the relationship between psychological factors and COVID-19 related anxiety after controlling for socio-demographic variables and early life stress (n = 189).**

| Model | R | *R^2^* | Adjusted *R^2^* | SE |
| --- | --- | --- | --- | --- |
| 1 | .240 | .057 | .032 | 4.05380 |
| 2 | .325 | .106 | .076 | 3.95911 |
| 3 | .663 | .439 | .411 | 3.16180 |
