# Supplementary material for: Psychological factors associated with COVID-19 related anxiety and depression in young adults during the COVID-19 pandemic
Source: PLoS One. 2023 Jun 2;18(6):e0286636. doi: 10.1371/journal.pone.0286636 (PMC10237641; doi:10.1371/journal.pone.0286636)
Supplement: S2 Table — (DOCX) [file pone.0286636.s002.docx]

**S2 Table. Hierarchical regression analysis of the relationship between psychological factors and COVID-19 related anxiety after controlling for socio-demographic variables and early life stress (n = 189).**

| Model | | Unstandardized Coefficients | | Standardized Coefficients | *t* | *p* | 95.0% Confidence Interval for B | | Collinearity Statistics | |
| --- | --- | --- | --- | --- | --- | --- | --- | --- | --- | --- |
|  |  | *B* | *SE* | *β* |  |  | Lower Bound | Upper Bound | Tolerance | VIF |
| 1 | (Constant) | -2.350 | 2.949 |  | -.797 | .427 | -8.169 | 3.469 |  |  |
|  | Age | .071 | .056 | .091 | 1.250 | .213 | -.041 | .182 | .967 | 1.034 |
|  | Sex | 1.642 | .638 | .197 | 2.572 | .011 | .382 | 2.901 | .878 | 1.139 |
|  | BMI | .120 | .072 | .129 | 1.665 | .098 | -.022 | .261 | .864 | 1.157 |
|  | SES | -.555 | .380 | -.107 | -1.461 | .146 | -1.304 | .194 | .959 | 1.043 |
|  | Education | .102 | 1.011 | .007 | .100 | .920 | -1.894 | 2.097 | .987 | 1.014 |
| 2 | (Constant) | -3.513 | 2.904 |  | -1.210 | .228 | -9.243 | 2.217 |  |  |
|  | Age | .044 | .056 | .057 | .788 | .432 | -.066 | .154 | .945 | 1.058 |
|  | Sex | 1.720 | .624 | .206 | 2.757 | .006 | .489 | 2.950 | .876 | 1.141 |
|  | BMI | .123 | .070 | .132 | 1.756 | .081 | -.015 | .262 | .864 | 1.157 |
|  | SES | -.442 | .372 | -.085 | -1.187 | .237 | -1.177 | .293 | .950 | 1.053 |
|  | Education | .389 | .992 | .028 | .393 | .695 | -1.568 | 2.347 | .978 | 1.022 |
|  | ETISR-SF | .240 | .077 | .226 | 3.140 | .002 | .089 | .391 | .951 | 1.052 |
| 3 | (Constant) | -4.364 | 2.505 |  | -1.742 | .083 | -9.308 | .579 |  |  |
|  | Age | -.006 | .046 | -.008 | -.135 | .893 | -.096 | .084 | .900 | 1.111 |
|  | Sex | 1.145 | .506 | .137 | 2.264 | .025 | .147 | 2.143 | .850 | 1.176 |
|  | BMI | .081 | .056 | .087 | 1.444 | .151 | -.030 | .192 | .859 | 1.164 |
|  | SES | -.152 | .310 | -.029 | -.489 | .625 | -.763 | .460 | .875 | 1.143 |
|  | Education | .343 | .796 | .024 | .430 | .667 | -1.228 | 1.914 | .968 | 1.033 |
|  | ETISR-SF | .089 | .069 | .083 | 1.278 | .203 | -.048 | .226 | .737 | 1.356 |
|  | 3-item RUL | -.019 | .112 | -.012 | -.171 | .864 | -.241 | .202 | .684 | 1.462 |
|  | FMPS | .007 | .013 | .038 | .564 | .573 | -.018 | .033 | .709 | 1.411 |
|  | WI-6 | .554 | .058 | .599 | 9.550 | .000 | .439 | .668 | .796 | 1.256 |
