# Supplementary material for: Psychological factors associated with COVID-19 related anxiety and depression in young adults during the COVID-19 pandemic
Source: PLoS One. 2023 Jun 2;18(6):e0286636. doi: 10.1371/journal.pone.0286636 (PMC10237641; doi:10.1371/journal.pone.0286636)
Supplement: S3 Table — (DOCX) [file pone.0286636.s003.docx]

**S3 Table. Model summary for the hierarchical regression analysis of the relationship between psychological factors and depression after controlling for socio-demographic variables and early life stress (n = 189).**

| Model | R | *R^2^* | Adjusted *R^2^* | SE |
| --- | --- | --- | --- | --- |
| 1 | .309 | .096 | .071 | 9.88950 |
| 2 | .493 | .243 | .218 | 9.07055 |
| 3 | .753 | .568 | .546 | 6.91475 |
