# Supplementary material for: Psychological factors associated with COVID-19 related anxiety and depression in young adults during the COVID-19 pandemic
Source: PLoS One. 2023 Jun 2;18(6):e0286636. doi: 10.1371/journal.pone.0286636 (PMC10237641; doi:10.1371/journal.pone.0286636)
Supplement: S4 Table — (DOCX) [file pone.0286636.s004.docx]

**S4 Table. Hierarchical regression analysis of the relationship between psychological factors and depression after controlling for socio-demographic variables and early life stress (n = 189).**

| Model | | Unstandardized Coefficients | | Standardized Coefficients | *t* | *p* | 95.0% Confidence Interval for B | | Collinearity Statistics | |
| --- | --- | --- | --- | --- | --- | --- | --- | --- | --- | --- |
|  |  | *B* | *SE* | *β* |  |  | Lower Bound | Upper Bound | Tolerance | VIF |
| 1 | (Constant) | 9.496 | 7.195 |  | 1.320 | .189 | -4.700 | 23.692 |  |  |
|  | Age | .126 | .138 | .066 | .916 | .361 | -.145 | .398 | .967 | 1.034 |
|  | Sex | 5.177 | 1.557 | .250 | 3.326 | .001 | 2.106 | 8.249 | .878 | 1.139 |
|  | BMI | .190 | .175 | .082 | 1.085 | .279 | -.156 | .536 | .864 | 1.157 |
|  | SES | -2.457 | .926 | -.191 | -2.654 | .009 | -4.285 | -.630 | .959 | 1.043 |
|  | Education | -1.387 | 2.467 | -.040 | -.562 | .575 | -6.255 | 3.481 | .987 | 1.014 |
| 2 | (Constant) | 4.434 | 6.654 |  | .666 | .506 | -8.694 | 17.562 |  |  |
|  | Age | .010 | .128 | .005 | .081 | .935 | -.242 | .262 | .945 | 1.058 |
|  | Sex | 5.517 | 1.429 | .266 | 3.861 | .000 | 2.698 | 8.337 | .876 | 1.141 |
|  | BMI | .206 | .161 | .089 | 1.279 | .203 | -.112 | .523 | .864 | 1.157 |
|  | SES | -1.968 | .853 | -.153 | -2.306 | .022 | -3.652 | -.284 | .950 | 1.053 |
|  | Education | -.135 | 2.273 | -.004 | -.059 | .953 | -4.619 | 4.350 | .978 | 1.022 |
|  | ETISR-SF | 1.045 | .175 | .394 | 5.961 | .000 | .699 | 1.392 | .951 | 1.052 |
| 3 | (Constant) | -3.204 | 5.479 |  | -.585 | .559 | -14.015 | 7.608 |  |  |
|  | Age | -.146 | .100 | -.076 | -1.465 | .145 | -.343 | .051 | .900 | 1.111 |
|  | Sex | 3.331 | 1.106 | .161 | 3.011 | .003 | 1.148 | 5.513 | .850 | 1.176 |
|  | BMI | .143 | .123 | .061 | 1.160 | .248 | -.100 | .385 | .859 | 1.164 |
|  | SES | -1.586 | .678 | -.123 | -2.340 | .020 | -2.924 | -.249 | .875 | 1.143 |
|  | Education | -1.513 | 1.741 | -.043 | -.869 | .386 | -4.949 | 1.923 | .968 | 1.033 |
|  | ETISR-SF | .256 | .152 | .096 | 1.685 | .094 | -.044 | .555 | .737 | 1.356 |
|  | 3-item RUL | 1.990 | .246 | .482 | 8.104 | .000 | 1.506 | 2.475 | .684 | 1.462 |
|  | FMPS | .061 | .029 | .124 | 2.130 | .035 | .004 | .118 | .709 | 1.411 |
|  | WI-6 | .525 | .127 | .228 | 4.137 | .000 | .274 | .775 | .796 | 1.256 |
